# Medically Refractory Nesidioblastosis as a Late Adverse Effect of Roux-en-Y Gastric Bypass

**DOI:** 10.7759/cureus.84429

**Published:** 2025-05-19

**Authors:** Michael Ladna

**Affiliations:** 1 Internal Medicine, University of California Davis Medical Center, Sacramento, USA

**Keywords:** hyperinsulinemic hypoglycemia, nesidioblastosis, non-insulinoma pancreatogenous hypoglycemia, recurrent syncope, roux-en-y gastric bypass (rygb)

## Abstract

A male in his late 40s with a past medical history of morbid obesity status post Roux-en-Y gastric bypass in 2004 presented to the emergency department with recurrent hypoglycemia. The hypoglycemic episodes were triggered by preceding hyperglycemia shortly after a meal. Due to the rapid drop in glucose, he often did not have sufficient time to ingest a rapid-acting carbohydrate snack, resulting in the progression of neuroglycopenic symptoms to syncope. His wife would then immediately administer intramuscular glucagon. A thorough workup did not reveal decompensated liver cirrhosis, chronic kidney disease, congestive heart failure, hypothyroidism, adrenal insufficiency, or insulin use. Serum insulin and C-peptide levels were profoundly elevated. A magnetic resonance imaging (MRI) of the abdomen and pelvis showed no pancreatic mass to suggest an insulinoma. He was referred to interventional radiology (IR) for a selective arterial calcium stimulation test (SACST), which showed an insulin ratio >2 in the gastroduodenal and hepatic arteries, consistent with a diagnosis of nesidioblastosis. He was trialed on numerous medications, which included octreotide, acarbose, diazoxide, and verapamil. He did not tolerate the octreotide due to the adverse effect of worsening abdominal pain and elevated serum lipase consistent with an attack of acute on chronic pancreatitis. The remaining medical regimen was ineffective at preventing hypoglycemia. Although evidence is lacking for use in this context, empagliflozin was then added to prevent the hyperglycemic spikes; however, this too proved ineffective at preventing hypoglycemic episodes. He underwent placement of a percutaneous endoscopic gastrostomy tube intended to tightly control his serum glucose via carbohydrate-low, protein-rich enteral feeds to prevent hyperglycemic episodes; however, this too failed due to suboptimal compliance with oral diet. Endocrinologic surgery declined distal pancreatectomy due to high morbidity and mortality risk with questionable benefit. The patient opted to seek a second opinion at another medical center.

## Introduction

There are several causes of hypoglycemia which include drug-induced (such as via the use of exogenous insulin), critical illness (including sepsis, hepatic dysfunction, and cardiac dysfunction), hormone deficiencies (adrenal insufficiency or hypothyroidism), or endogenous overproduction of insulin which is also known as pancreatogenic hyper-insulinemic hypoglycemia (PHH). In the adult population, the majority (over 90%) of PHH cases are due to an insulinoma, a functional tumor arising from the beta-cells of the islets of Langerhans [1]. Nesidioblastosis is not as well defined as insulinomas due to their rarity but has been reported in association with bariatric surgery, especially status post-Roux-en-Y gastric bypass [2]. Nesidioblastosis is classified as a non-insulinoma pancreatogenous hypoglycemia (NIPH) and accounts for only 0.5 to 5% of cases of PHH [3]. 

## Case presentation

A male in his 40s with a past medical history of morbid obesity status post Roux-en-Y gastric bypass in 2004 complicated by a marginal ulcer, splenic artery aneurysm status post clipping, chronic pancreatitis (CP), and opiate dependency from chronic pain related to CP, presented to the emergency department (ED) multiple times for recurrent rapid hypoglycemia resulting in syncope. The hypoglycemic episodes were postprandial and did not occur during fasting or exercise. There was no history of alcoholism, liver cirrhosis, chronic kidney disease (CKD), congestive heart failure (CHF), thyroid disease, or adrenal insufficiency. The workup revealed unremarkable liver function tests (LFT), creatinine, blood urea nitrogen (BUN), B-type natriuretic peptide (BNP), thyroid-stimulating hormone (TSH), free thyroxine (T4), and morning cortisol levels. A continuous glucose monitor (CGM) showed that each episode of hypoglycemia was preceded by serum glucose > 140mg/dL and often > 180mg/dL. Due to the hypoglycemia developing so rapidly, he was unable to consume a carbohydrate-rich snack to prevent a syncopal episode. His wife quickly provided him with glucagon IM due to his symptoms. He reported a fear of leaving his home and a tendency to avoid eating for fear of triggering hypoglycemia. He would often fast until he became very hungry and then would end up eating a carbohydrate-dense meal, resulting in an over-aggressive insulin response with resultant severe hypoglycemia. As such, he reported a significant deterioration in his quality of life and the development of depression, though without suicidal ideation.

Endocrinology was consulted for assistance with workup and management of hypoglycemia. The initial workup for hypoglycemia revealed an elevated insulin level of 182.8 uU/mL (ref range: 2.0 - 22.1 uU/mL), an elevated C-peptide level of 17.9 ng/mL (ref range: 0.5 - 3.3 ng/mL), and an undetectable insulin antibody level of less than 0.4 U/mL (ref range: 0.0 - 0.4 U/mL). A 72-hour fasting hypoglycemia test did not result in hypoglycemia, which was defined as plasma glucose less than 55mg/dL with symptoms of hypoglycemia or plasma glucose less than 45mg/dL without symptoms of hypoglycemia. Immediately following the 72-hour fasting hypoglycemia test, a mixed meal test was performed, which consisted of having the patient fast for 10 hours overnight and then consume 300 mL of a mixed-meal drink containing 450 kcal and 60g of CHO. The mixed meal test did provoke hypoglycemia. Per endocrinology recommendations, he was instructed to eat a low-carbohydrate diet to minimize the risk of post-prandial hyperglycemia and started on octreotide. With the use of octreotide, he had adverse symptoms that were hardly tolerable, which included worsening abdominal pain, watery diarrhea, and an elevated serum lipase, suggesting an acute-on-chronic pancreatitis attack. During the short time he was compliant with octreotide, he continued to have episodes of hypoglycemia. Due to adverse effects and lack of efficacy, octreotide was stopped. He was then trialed on a combination of acarbose 100mg three times daily (TID), diazoxide 120mg TID, and verapamil 120mg twice daily (BID). Despite reported compliance with dietary restrictions and the medical regimen, he continued to have post-prandial hypoglycemia with further visits to the ED. Empagliflozin 10mg daily was then initiated to prevent post-prandial hyperglycemia, since this was the trigger for hypoglycemia. Empagliflozin 10mg daily was not effective, and thus the dose was increased to 25mg daily; however, this too proved ineffective at preventing the trigger of post-prandial hyperglycemia. A computed tomography (CT) of the abdomen and pelvis, followed by a magnetic resonance image (MRI) of the abdomen and pelvis, did not show any pancreatic masses to suggest a neuroendocrine tumor such as insulinoma. He was referred to interventional radiology (IR) for a selective arterial calcium stimulation test (SACST).

**Figure 1:**
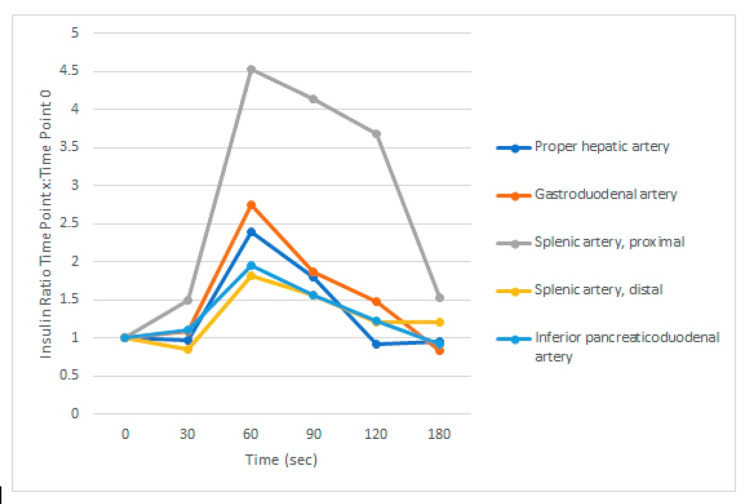
Insulin ratio at baseline and 30, 60, 90, 120, 180 seconds after calcium gluconate stimulation

The SACST is a diagnostic test that involves the selective injection of calcium gluconate into the arteries supplying the pancreas, followed by subsequent hepatic venous sampling at various time points from 30 to 180 seconds at 30-second intervals from calcium injection to measure insulin levels in those arteries. The rationale for this test is that calcium stimulates insulin production from insulinoma and nesidioblastosis cells but not from normal beta-cells. This test allows for both diagnosis and precise localization of insulinoma. The increase in insulin considered diagnostic for an insulinoma or nesidioblastosis is an insulin ratio of >2. If this ratio occurs in a single artery, it is diagnostic of insulinoma; however, if an insulin ratio >2 is measured in multiple arteries, this is diagnostic of nesidioblastosis. The SACST showed an insulin ratio >2 in the proximal splenic artery, gastroduodenal artery (GDA), and proper hepatic artery (PHA), consistent with a diagnosis of nesidioblastosis (Table 1, Figure 1). Figure 1 shows the relative insulin levels of the five arteries investigated, with the proximal splenic artery showing an insulin ratio peak of 4.5, the GDA showing an insulin ratio peak of approximately 2.8, and the PHA having an insulin ratio peak of 2.4 at the 60-second mark from injection of calcium. Table 1 shows the absolute and relative insulin levels with time 0 indicating administration of calcium gluconate, followed by measures of insulin level at arteries of interest at 30, 60, 90, 120, and finally 180 seconds. 

**Table 1:** Absolute and relative insulin concentrations measured during SACST measured at baseline levels and then at 30, 60, 90, 120, 180 seconds after calcium gluconate stimulation

Time (second)	ABSOLUTE INSULIN LEVEL (uU/mL)					RELATIVE INSULIN LEVEL				
	Proper hepatic artery	Gastro duodenal artery	Splenic artery, proximal	Splenic artery, distal	PDA	Proper hepatic artery	Gastro duodenal artery	Splenic artery, proximal	Splenic artery, distal	PDA
0	4.0	4.4	3.4	3.4	5.0	1	1	1	1	1
30	3.9	4.8	5.1	2.9	5.5	0.98	1.09	1.50	0.85	1.10
60	9.6	12.1	15.4	6.2	9.8	2.40	2.75	4.53	1.82	1.96
90	7.2	8.2	14.1	5.3	7.8	1.80	1.86	4.15	1.56	1.56
120	3.7	6.5	12.5	4.1	6.1	0.93	1.48	3.68	1.21	1.22
180	3.8	3.7	5.2	4.1	4.6	0.95	0.84	1.53	1.21	0.92

He was then referred to endocrine surgery with a tentative plan for distal pancreatectomy; however, in light of the high morbidity and limited efficacy of surgical interventions for nesidioblastosis, the decision was made to defer surgery in favor of a less invasive approach. He received a G-tube with the hope that tight control of carbohydrate intake via carbohydrate-low enteral feeds and the continuation of the medical regimen would prevent post-prandial hyperglycemic triggers. Despite this intervention, he continued to be admitted for syncope due to recurrent post-prandial hypoglycemia. It was discovered he was not fully compliant with a carbohydrate-restricted diet and would have carbohydrate-rich snacks along with his enteral feeds. Endocrinology was re-engaged, and recommended initiation of pasireotide. Subcutaneous pasiroeotide was initiated at 0.9mg/mL twice daily during another hospitalization for hypoglycemia; however, despite multiple efforts by pharmacy and his primary care physician was not able to get prior authorization from the patient's insurance to cover this medication, and the patient was unable to afford the medication without insurance coverage. Endocrine surgery was re-engaged and ultimately declined to proceed with distal pancreatectomy due to concern for complications (including worsening his chronic abdominal pain), with suboptimal success rates with this intervention per available literature. The patient was dissatisfied with this conclusion and sought a second opinion at another medical center.

## Discussion

Although data on the incidence rate of nesidioblastosis are scarce, it is estimated to occur in 0.1 to 0.3% of Roux-en-Y gastric bypasses [4]. Nesidioblastosis typically presents months to years after index surgery and is classified as a rare late adverse effect of bariatric surgery [5]. The annual incidence of adult-onset nesidioblastosis is believed to be less than 0.1 in 1,000,00,0, with a mean age of onset of 47 years [6]. Nesidioblastosis after Rou-en-Y bypass, as was the case in our patient, is hypothesized to be due to a reactive process that occurs in response to metabolic and hormonal changes from substantial weight loss after gastric bypass surgery [7]. One proposed mechanism is related to the observation that patients with post-RYGB hypoglycemia have significantly higher levels of glucose-dependent insulinotropic polypeptide (GIP) and glucagon-like peptide-1 (GLP-1), both of which exert a hypertrophic effect on the pancreatic islet cells. Nesidioblastosis is characterized by pancreatic beta-cell hypertrophy, islet hyperplasia, and increased beta-cell mass [8].

To solidify a diagnosis of nesidioblastosis, a clinician must first rule out insulinoma, autoimmune causes of hyperinsulinemia, and factitious hyperinsulinemia. The diagnosis of nesidioblastosis should be considered when cross-sectional imaging (CT or MRI) is negative for an insulinoma and there is a positive SACST. The SACST is based on the observation that calcium stimulates insulin release from hyperfunctioning, abnormal pancreatic beta-cells but not from normal pancreatic beta-cells. In nesidioblastosis, SACST should show a diffuse pattern with doubling or tripling of the basal hepatic venous serum insulin concentration in multiple arteries, whereas an insulinoma is typically associated with a focal secretion pattern in a single artery [4]. Although SACST is the most specific non-invasive test, histopathology is the gold standard for diagnosing nesidioblastosis. This is in part due to the finding that 75% of patients with nesidioblastosis and 25% of patients with insulinoma demonstrated positivity in two or more pancreatic arterial distributions on SACST, making it difficult to differentiate between the two pathologies via the results of SACST alone [9]. However, pancreatic resection and biopsy are not always feasible, as in this case. As such, a diagnosis of nesidioblastosis can be made from the history of hyperinsulinemic post-prandial hypoglycemia, lack of pancreatic mass on cross-sectional imaging, and SACST showing a diffuse pattern of insulin release. Even if there are two or more pancreatic arterial distributions, the degree of insulin elevation can aid in differentiating an insulinoma from nesidioblastosis, since insulinomas tend to release higher amounts of insulin into the circulation. The maximum hepatic venous insulin concentration (mHVI) and relative fold hepatic venous insulin concentration (rHVI) were significantly higher in an insulinoma compared to nesidioblastosis. mHVI cut-offs of >9.5 and >263.5 ulU/mL were 95 and 100% specific for insulinoma, respectively, while a 19-fold increase in rHVI over baseline was 99% specific for insulinoma [9]. This case presented with a significantly higher insulin ratio in the proximal splenic artery compared to the other arteries; however, the maximum insulin level of 15.4 uU/mL (insulin ratio of 4.53) is still quite low compared to the insulin level increases typically seen in insulinomas [9]. In addition, the lack of pancreatic mass on MRI makes this diagnostic workup more consistent with nesidioblastosis and not insulinoma. 

A novel non-invasive modality for the diagnosis of PHH is based on the observation that insulinoma beta-cells have a higher density of glucagon-like peptide-1 (GLP-1) receptors (GLP-1R) [10]. Several novel radiotracers have been developed that target this receptor (indium-11, technetium-99m, and gallium-68-labeled exendin-4) [9]. A recent prospective study showed that the sensitivity of Ga-DOTA-extendin-4 positron emission tomography (PET) CT for detecting insulinomas was 94.6% [11]. This test also yields the promise of differentiating between insulinoma and nesidioblastosis since the density of GLP-1R in nesidioblastosis is higher than in normal beta-cells but lower than in an insulinoma [10]. Further trials are required to determine if Ga-DOTA-extendin-4 PET/CT can indeed effectively differentiate between insulinomas and nesidioblastosis.

Medical management and dietary modification remain the first-line therapeutic modalities for the treatment of nesidioblastosis. Patients with nesidioblastosis have been successfully treated with a combination of diazoxide and dietary measures, which consisted of regular intake of complex carbohydrates with slow absorption throughout the day [12]. Medical management options include the use of a somatostatin analog such as octreotide, acarbose, and verapamil; however, treatment responses are highly variable [13,14].

Although surgical options do exist, with case reports and case series documenting successful treatment of nesidioblastosis via surgical enucleation [15], near-total pancreatectomy [16], and distal pancreatectomy [17], these interventions carry significant morbidity and mortality rates. Although individual case reports have reported clinical success with pancreatectomy, the largest retrospective study of patients with non-insulinoma pancreatogenous hypoglycemia (NIPH) carried out at the Mayo Clinic revealed conflicting results. A total of 75 patients with NIPH underwent partial pancreatectomy from 1996 to 2008. Of those 75 patients, 48 patients completed the quality of life (EQ-5D) and fear of hypoglycemia scale (FOHS) surveys. The investigators found that 41/48 patients (87%) had a recurrence of hypoglycemia at a median time of 16 months after partial pancreatectomy. A total of 36 (75%) of the patients who had recurrence still reported an improvement in quality of life and symptoms as reflected by a statistically significant increase in median EQ-5D score from 40 to 75 out of 100 (P<0.001) and reduction in psychologic stress from neuroglycopenic symptoms with greater than 50% decrease in FOHS (P<0.001). A total of 25% of patients experienced no benefit after pancreatectomy [18]. A study that included five patients with nesidioblastosis treated with distal pancreatectomy found that three (60%) patients achieved euglycemia while two (40%) patients had a recurrence of hypoglycemia and were then successfully managed with the initiation of verapamil [19]. Another study that looked at 15 patients with nesidioblastosis found that eventually, nine (60%) of these patients required surgical intervention in the form of distal pancreatectomy. Although all patients initially reported marked relief of symptoms, only two (22.2%) patients had sustained resolution of symptoms (at a median follow-up of 22 months, range of 8-54 months). Three (33.3%) developed occasional symptoms (1-2 times per month) which did not require any medications, two developed frequent symptoms (>2 times per month) which were effectively controlled via the addition of medications, and two patients had severe symptoms refractory to any medical therapy (calcium channel blocker [CCB], diazoxide, octreotide) [20]. Since larger retrospective studies with meaningful follow-up times showed suboptimal clinical success rates with pancreatectomy along with significant morbidity and mortality associated with such an aggressive surgical approach, it should be reserved as a last resort for the management of nesidioblastosis.

## Conclusions

In conclusion, nesidioblastosis is a rare and poorly understood late adverse event of Roux-en-Y gastric bypass associated with significant morbidity and poor quality of life. Due to mimicking insulinomas, this condition can be especially difficult to diagnose, and there is often a delay in the diagnosis, with a subsequent delay in the initiation of appropriate treatment. Although nesidioblastosis can be effectively managed with dietary measures and medical therapy alone, some cases are refractory, as was the situation in the case presented above. For this subset of patients, surgical intervention in the form of pancreatectomy can be considered; however, given the lack of effectiveness and significant morbidity and mortality associated with such a demanding surgery, all other therapeutic modalities should be exhausted first. Although our patient had exhausted medical management, the risks of surgical intervention outweighed potential benefits per endocrinologic surgery evaluation, and as such, pancretectomy was not pursued. Clinicians should be aware of this diagnosis when evaluating an adult patient presenting with hyperinsulinemic hypoglycemia with a history of Roux-en-Y gastric bypass and be familiar with how to differentiate nesidioblastosis from the more common insulinoma.
